# Involvement of a PadR regulator PrhP on virulence of *Ralstonia solanacearum* by controlling detoxification of phenolic acids and type III secretion system

**DOI:** 10.1111/mpp.12854

**Published:** 2019-08-08

**Authors:** Yong Zhang, Weiqi Zhang, Liangliang Han, Jing Li, Xiaojun Shi, Yasufumi Hikichi, Kouhei Ohnishi

**Affiliations:** ^1^ College of Resources and Environment Southwest University Chongqing China; ^2^ Key Laboratory of Efficient Utilization of Soil and Fertilizer Resources Chongqing; ^3^ Research Institute of Molecular Genetics, Kochi University Kochi Japan; ^4^ The Ninth Peoples Hospital of Chongqing Chongqing China; ^5^ Laboratory of Plant Pathology and Biotechnology Kochi University Kochi Japan

**Keywords:** degradation of phenolic acid, PadR regulator, pathogenesis, *Ralstonia solanacearum*, type III secretion system

## Abstract

*Ralstonia solanacearum* can metabolize ferulic acid (FA) and salicylic acid (SA), two representative phenolic acids, to protect it from toxicity of phenolic acids. Here, we genetically demonstrated a novel phenolic acid decarboxylase regulator (PadR)‐like regulator PrhP as a positive regulator on detoxification of SA and FA in *R. solanacearum*. Although the ability to degrade SA and FA enhances the infection process of *R. solanacearum* toward host plants, PrhP greatly contributes to the infection process besides degradation of SA and FA. Our results from the growth assay, promoter activity assay, RNA‐seq and qRT‐PCR revealed that PrhP plays multiple roles in the virulence of *R. solanacearum*: (1) positively regulates expression of genes for degradation of SA and FA; (2) positively regulates expression of genes encoding type III secretion system (T3SS) and type III effectors both *in vitro* and *in planta*; (3) positively regulates expression of many virulence‐related genes, such as the flagella, type IV pili and cell wall degradation enzymes; and (4) is important for the extensive proliferation *in planta*. The T3SS is one of the essential pathogenicity determinants in many pathogenic bacteria, and PrhP positively regulates its expression mediated with the key regulator HrpB but through some novel pathway to HrpB in *R. solanacearum*. This is the first report on PadR regulators to regulate the T3SS and it could improve our understanding of the various biological functions of PadR regulators and the complex regulatory pathway on T3SS in *R. solanacearum*.

## Introduction

Phenolic acids are abundant in the plant kingdom and are involved in the structure of plant cell walls. They belong to the class of secondary metabolites and bioactive compounds synthesized by plants (Verpoorte *et al.*, 2002; Goleniowski *et al*., 2013). Phenolic compounds basically act as signalling molecules in the interaction between plants and microbes. In fact, they play a crucial role in initiating the symbiotic relationship between plants and useful microorganisms. Moreover, they usually disrupt membrane integrity and decouple the respiratory proton gradient, and hence are broadly antimicrobial (Fitzgerald *et al.*, 2004; Harris *et al.*, 2010). Hydroxybenzoic acids (typically as salicylic acid, SA) and hydroxycinnamic acids (HCAs, typically as ferulic acid, FA) are two important types of natural phenolic acids (Goleniowski *et al*., 2013; Heleno *et al.*, 2015). Several lines of evidence suggest that phenolic acids are involved in the interaction between host plants and pathogens (Campos *et al.*, 2014; Fry *et al.*, 2000; Naoumkina *et al.*, 2010). Many plants can release de novo synthesized HCAs into the rhizosphere in response to pathogen infection (Lanoue *et al.*, 2010; Wallis and Chen, 2012). In order to protect themselves from the toxicity of phenolic acids, many bacterial species express phenolic acid decarboxylase (PADse, typically as *padC* gene product in *Bacillus subtilis*) to convert phenolic acids into less toxic 4‐vinyl and 4‐ethyl derivatives (Park *et al.*, 2017; Tran *et al.*, 2008). Phenolic acid decarboxylase regulators (PadRs) are a large group of transcriptional regulators that promote the PADse‐mediated detoxification of phenolic acids in many bacterial species. They can usually initiate the expression of PADse genes by release from their promoters in the presence of phenolic acids since they usually bind to promoters of PADse genes in the absence of phenolic acids and repress their expression (Florez *et al.*, 2015; Huillet *et al.*, 2006; Nguyen *et al.*, 2011). The PadR regulators have been characterized to function in various survival processes of detoxification, antibiotic biosynthesis, multidrug resistance, toxin production and carbon catabolism (Florez *et al.*, 2015; Huillet *et al.*, 2006; Nguyen *et al.*, 2011; Tran *et al.*, 2008). Moreover, some bacteria, including *Pseudomonas* sp., *Acinetobacter calcoaceticus* and *Ralstonia solanacearum*, can protect themselves from the toxicity of phenolic acids by degrading them as carbon sources for metabolism at moderate concentrations (Gasson *et al.*, 1998; Lowe‐Power *et al.*, 2018; Overhage *et al.*, 1999; Segura *et al.*, 1999).

*Ralstonia solanacearum* is the causal agent of bacterial wilt disease in over 450 plant species of 50 botanical families worldwide (Jiang *et al.*, 2017; Vasse *et al.*, 1995). The ability to degrade SA and HCAs can facilitate its infection process in host plants to some extent (Lowe *et al.*, 2015; Lowe‐Power *et al*., 2016). The bacterium usually remains invasive at moderate concentrations of these phenolic chemicals but just survives at higher concentrations. Different from the PADse‐mediated detoxification, degradation of the HCAs and SA in *R. solanacearum* is dependent on the *fca‐fcs* operon and *nag* operon, respectively. The *fcs* operon encodes a feruloyl‐CoA synthetase that converts HCAs into phenolic aldehydes, while the *nag* operon encodes dioxygenases that convert SA into double hydroxyl‐benzoic acid, which is unstable and causes the aromatic ring to open (Gasson *et al.*, 1998; Lowe *et al.*, 2015; Lowe‐Power *et al.*, 2016). To date, a total of 9000 PadR members have been deposited to the Pfam database, while no PADse can be annotated in genomes of abundant *R. solanacearum* strains (https://iant.toulouse.inra.fr/bacteria/annotation/cgi/ralso.cgi). The regulation mechanism of degradation of phenolic acids remains to be further elucidated in *R. solanacearum*.

As a soil‐borne, vascular bacterium, *R. solanacearum* generally invades host plants through natural root openings or root wounds (Janse *et al.*, 2004; Vasse *et al.*, 1995). Once it invades the xylem vessels, it proliferates extensively and produces a large amount of exopolysaccharides (EPS) to block sap flow in the xylem vessels, causing plants to rapidly stunt and wilt (Denny, 1995; Roberts *et al.*, 1988). In addition to the EPS, a syringe‐like type III secretion system (T3SS) is essential for the pathogenicity, and the bacteria use this to inject virulence factors (type III effectors, T3Es) into the host cytosol to subvert the host defence (Angot *et al.*, 2006; Cunnac *et al.*, 2004; Jones and Dangl, 2006). The T3SS is greatly conserved among numerous *R. solanacearum* strains, which is encoded by approximately 20 genes of the hypersensitive response and pathogenicity (*hrp*) regulon and is directly controlled by a master regulator HrpB, an AraC family of transcriptional regulator (Arlat *et al.*, 1992; Coll and Valls, 2013; Mukaihara *et al.*, 2010). Expression of the T3SS, *hrpB* and T3Es is not activated until the bacterium has contact with host signals or some mimic signals, such as those in nutrient‐limited medium that might mimic the plant apoplastic fluids (Marenda *et al.*, 1998; Yoshimochi *et al.*, 2009; Zhang *et al.*, 2013). Expression of *hrpB* and T3SS has been well demonstrated to be globally regulated by a complex network including dozens of regulators, such as HrpG and PrhG, the PrhA‐PrhR/I‐PrhJ‐HrpG signalling cascade and some other well‐characterized regulators of PhcA, PrhN, PrhO and XpsR (Genin and Denny, 2012; Hikichi *et al.*, 2017; Valls *et al.*, 2006; Zhang *et al.*, 2018). HrpG and PrhG are close paralogues of the OmpR/PhoB family of two‐component response regulators that respond to host signals by phosphorylation and activate the *hrpB* expression in a parallel way (Plener *et al.*, 2010; Zhang *et al.*, 2013). Host signals or mimic signals are presumed to be recognized by an outer membrane protein PrhA and transferred to HrpG via the PrhA‐HrpG signalling cascade, which begins to activate *hrpB* expression (Genin and Denny, 2012; Hikichi *et al.*, 2017; Valls *et al.*, 2006; Zhang *et al.*, 2018). Moreover, *R. solanacearum* integrates numerous virulence factors, including the flagella, type IV pili and cell wall degradation enzymes (CWDEs) to promote its infection process toward host plants (Genin and Denny, 2012; Hikichi *et al.*, 2007).

In order to further elucidate the global regulation of the T3SS in *R. solanacearum*, we generated a *popA‐lacZYA* fusion to monitor expression profiles of the T3SS in a Japanese *R. solanacearum* OE1‐1 and screened several T3SS‐regulating candidates with transposon mutagenesis. The *popA* gene is located on the left side of the *hrp* regulon that belongs to T3Es and is directly controlled by HrpB. The *popA‐lacZYA* exhibits an identical expression prolife to the *hrp* regulon under different conditions. Moreover, this fusion does not affect the infection process of OE1‐1 toward host plants. Among them is Rsp0309, which is annotated as a putative PadR‐like regulator (Zhang *et al.*, 2013). The PadR regulators are known to be involved in various cellular processes, including virulence but not the T3SS. We thus focused on the Rsp0309, hereafter designated PrhP, to investigate its regulatory roles on the detoxification of phenolic acids, regulation of the T3SS and contribution to pathogenicity of *R. solanacearum*.

## Results

### PrhP is important for detoxification of SA and FA in *R. solanacearum*

**Figure 1:**
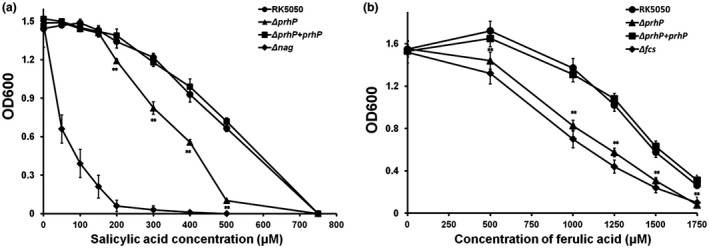
Growth of OE1‐1 derivates in nutrient‐rich medium with supplementary (a) salicylic acid (SA) and (b) ferulic acid (FA) at different concentrations. Strains were cultivated at 28 °C and OD_600_ was measured at about 15 h post‐inoculation (hpi). RK5050 refers to the wild‐type *Ralstonia solanacearum* strain, the *ΔprhP* refers to RQ5649 (RK5050, *ΔprhP*) and the *ΔprhP* + *prhP* refers to RQ5649 with complementary PrhP_OE1‐1_. The Δ*nag* refers to RQ6058 (RK5050, Δ*nagAL*) and Δ*fcs* refers to RQ5625 (RK5050, Δ*fcs*). Mean values of four biological replicates were averaged and presented with SD (error bars). Statistical significance between RQ5649 and RK5050 was assessed using a post hoc Dunnett test following ANOVA. Significance level: ** indicates *P* < 0.01.

The PadR regulators are well known to promote the detoxification of phenolic acids in many bacterial species (Gury *et al.*, 2004, 2009). We thus generated a *prhP* mutant (RQ5649) to ascertain whether *R. solanacearum* PrhP is involved in the detoxification of SA and FA, two representative phenolic acids. Although growth of the wild‐type strain (RK5050) and RQ5649 was severely impaired with increasing concentration of supplementary SA and FA in both nutrient‐rich (Fig. 1a,b) and nutrient‐limited media (*hrp*‐inducing medium, data not shown), the *prhP* mutant was much more susceptible to SA and FA than the wild‐type strain, especially at low concentrations (Fig. 1a,b). Degradation of SA and FA is validated to be dependent on operons of *nag* and *fcs* in *R. solanacearum*, respectively (Lowe *et al.*, 2015; Lowe‐Power *et al*., 2016), and we generated a *nag* mutant (RQ6058) and an *fcs* mutant (RQ5625) to compare their susceptibility with the *prhP* mutant. The *nag* mutant was substantially more susceptible than the *prhP* mutant to SA in rich medium (Fig. 1a), while the *fcs* mutant exhibited similar susceptibility as the *prhP* mutant to FA in rich medium (Fig. 1b). The complementary *prhP_OE1‐1_* fully restored the impaired growth of the *prhP* mutant to that of the wild‐type strain in medium with supplementary SA and FA (Fig. 1a,b), confirming that PrhP is important for the detoxification of SA and FA in *R. solanacearum*.

### PrhP positively regulates expression of genes for SA degradation

**Figure 2:**
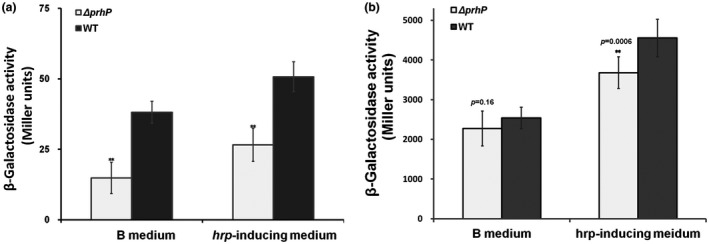
Expression of *nagAa‐lacZYA* in *prhP* mutants in nutrient‐rich and nutrient‐limited (*hrp*‐inducing) media (a) without salicylic acid (SA) and (b) with supplementary SA at a concentration of 250 μM. The WT refers to wild‐type *Ralstonia solanacearum* RQC607 (OE1‐1, *nagAa‐lacZYA*) and the Δ*prhP* refers to RQC609 (OE1‐1, Δ*prhP*, *nagAa‐lacZYA*). Cells were grown in medium to an OD_600_ of about 0.1 and subjected for the β‐galactosidase assay. Enzymatic activities are presented in Miller units (Miller, 1992). Mean values of four biological replicates were averaged and presented with SD (error bars). Statistical significance between RQC609 and RQC607 was assessed using a post hoc Dunnett test following ANOVA. Significance level: ** indicates *P* < 0.01.

The PadR regulators usually bind to promoters of PADse genes in the absence of phenolic acids to repress their expression, while they initiate the expression of PADse genes by release from their promoters in the presence of phenolic acids (Florez *et al.*, 2015; Huillet *et al.*, 2006; Nguyen *et al.*, 2011). We generated a *nagAa‐laZYA* reporter fusion to monitor the expression of the *nag* operon and ascertained how PrhP regulates *nag* expression in conditions with or without supplementary SA. Consistent with a previous report (Lowe‐Power *et al.*, 2016), supplementary SA greatly enhanced *nag* expression in RQC607 (OE1‐1, *nagAa‐lacZYA*), which reached to about 50‐ and 100‐fold higher levels in nutrient‐rich and nutrient‐limited media, respectively (Fig. 2a,b). Although *nag* expression remains at quite a low level in the absence of SA, it was substantially decreased in RQC609 (OE1‐1, Δ*prhP*, *nagAa‐lacZYA*) in both media, and the complementary *prhP*_OE1‐1_ fully restored the impaired *nag* expression to that of the parent strain (RQC607) (Fig. 2a). In the presence of SA, *nag* expression was significantly reduced with *prhP* deletion in nutrient‐limited medium (*P* = 0.0006), but there was no alteration in nutrient‐rich medium (*P* = 0.16) (Fig. 2b). It is worthwhile noting that the *nagAa* expression in the *prhP* mutant is slightly decreased to about 80% of levels of the parent strain (RQC607) in nutrient‐limited medium (Fig. 2b).

### PrhP positively regulates the T3SS expression both *in vitro* and *in planta*

**Figure 3:**
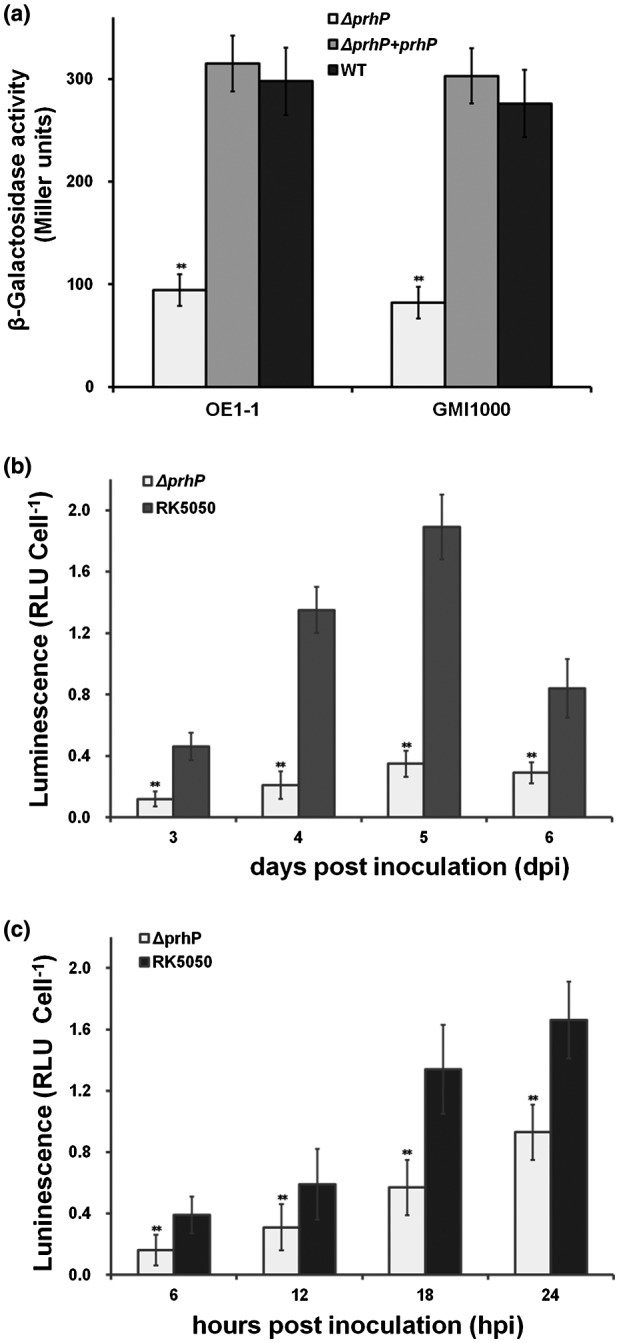
Expression of *popA* in different *prhP* mutants (a) *prhP* mutants from *Ralstonia solancearum* OE1‐1 and GMI1000 in *hrp*‐inducing medium, (b) *prhP* mutants from OE1‐1 in tomato stems, (c) *prhP* mutants from OE1‐1 in tobacco leaves. The WT refers to the wild‐type strain of RK5050 (OE1‐1, *popA‐lacZYA*) and GF0001(GMI1000, *popA‐lacZYA*). The Δ*prhP* from OE1‐1 refers to RQ5649 (RK5050, Δ*prhP*) and that from GMI1000 refers to GF0018 (GMI1000, *popA‐lacZYA*, Δ*prhP*). The Δ*prhP* + *prhP* from OE1‐1 refers to RQ5649 with complementary PrhP_OE1‐1_ and that from GMI1000 refers to GF0018 with complementary PrhP_OE1‐1_. (a) Cells were cultivated to an OD_600_ of approximately 0.1 and subjected for β‐galactosidase assay. Enzymatic activities are presented in Miller units. (b) Tomato plants were inoculated with petiole inoculation, which started wilting at about 3 days post‐inoculation (dpi) and died at about 6 dpi. Stem species were thus removed at 3–6 dpi and cells were harvested for the enzyme assay *in planta* with the Galacto‐Light Plus kit. (c) Tobacco leaves were infiltrated with cells suspension of about 0.1 OD_600_ and leaf disks were punched every 6 h for the enzyme assay *in planta*. Enzymatic activity was presented with luminescence normalized with cells numbers. Luminescence was evaluated using the GloMax20 luminometer (Promega) and cells numbers were quantified by dilution plating. Mean values of four biological replicates were averaged and presented with SD (error bars). Statistical significance between the Δ*prhP* and the parent strain was assessed using a post hoc Dunnett test following ANOVA. Significance level: ** indicates *P* < 0.01.

The PrhP was originally screened as a T3SS‐regulating candidate by transposon mutagenesis, in which the *popA‐lacZYA* fusion was constructed to monitor the T3SS expression in *R. solanacearum* (Zhang *et al.*, 2013). Consistent with those in transposon mutants, *popA* expression was significantly reduced in the *prhP* mutant (94 versus 256 Miller units of the wild‐type strain) in *hrp*‐inducing medium, and the complementary *prhP*_OE1‐1_ completely restored its reduced *popA* expression to that of the wild‐type strain (Fig. 3a).

*Ralstonia solanacearum* is greatly heterogeneous and different strains usually exhibit different phenotypes even in the same host. For instance, the standard strain GMI1000 is avirulent in tobacco plants, while the Japanese strain OE1‐1 is virulent in tobacco plants. We therefore generated a *prhP* mutant (GF0018) from GF0001 (GMI1000, *popA‐lacZYA*) to ascertain whether PrhP regulates the T3SS expression in different *R. solanacearum* strains. Consistent with that from OE1‐1, *popA* expression was significantly impaired in the *prhP* mutant (GF0018), and the complementary PrhP_OE1‐1_ fully restored the reduced *popA* expression in GF0018 to that of the parent strain (GF0001) (Fig. 3a), confirming that the PrhP of OE1‐1 is functionally equivalent to that of GMI1000 and the regulation of PrhP on T3SS is conserved in different *R. solanacearum* strains.

The T3SS expression can be enhanced to a much higher level *in planta* than in *hrp*‐inducing media (Yoshimochi *et al.*, 2009; Zhang *et al.*, 2013). We thus recovered bacterial cells from tomato stems and tobacco leaves to ascertain whether PrhP is required for T3SS expression *in planta*. The wild‐type strain (RK5050) usually withers and kills petiole‐inoculated tomato plants at 3 and 7 days post‐inoculation (dpi), respectively. We thus recovered bacterial cells from petiole‐inoculated tomato stems at 3–6 dpi and subjected them for the enzyme assay. The *prhP* mutant (RQ5649) exhibited significantly impaired *popA* expression in tomato stems compared with the wild‐type strain at 3–6 dpi (Fig. 3b). Bacterial cells were also recovered from tobacco leaves, which were infiltrated with cell suspension at an OD_600_ of about 0.1, at 6–24 h post‐infiltration (hpi) and subjected for the enzyme assay. The *prhP* deletion significantly impaired the *popA* expression in tobacco leaves at 6–24 hpi (Fig. 3c). All these results confirmed that PrhP positively regulates T3SS expression both *in vitro* and *in planta*.

### PrhP greatly promotes infection process of *R. solanacearum* besides degradation of SA and FA

**Figure 4:**
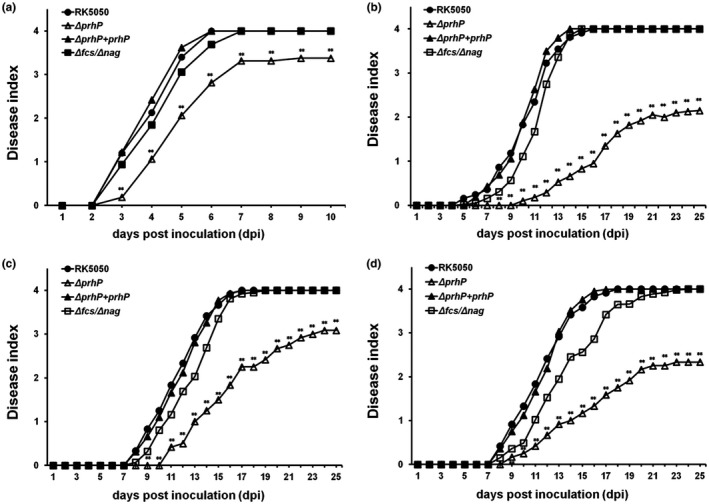
Virulence assay of *Ralstonia solanacearum* *prhP* mutants in (a) tomato plants with petiole inoculation, (b) tomato plants with soil‐soaking inoculation, (c) tobacco plants with leaf infiltration and (d) tobacco plants with soil‐soaking inoculation. The Δ*fcs*/Δ*nag* refers to RQ6207 (RK5050, Δ*fcs,* Δ*nag*). For the soil‐soaking inoculation, a bacterial suspension was poured into pot soil of plants at a final concentration of 10^7^ cfu/g of soil. For the petiole inoculation, 3 µL of bacterial suspension at 10^8^ cfu/mL was dropped onto the freshly cut surface of petioles. For the leaf infiltration, about 50 µL of bacterial suspension at 10^8^ cfu/mL was infiltrated into tobacco leaves with a blunt‐end syringe. Wilt symptoms were inspected daily and scored on a disease index scale from 0 to 4 (0, no wilting; 1, 1–25% wilting; 2, 26–50% wilting; 3, 51–75% wilting; and 4, 76–100% wilted or dead). Each assay was repeated at least with four biological replicates and each trial contained at least 12 plants. Mean values of all results were averaged and presented with SD (error bars), but the SD is not presented in figures for aesthetic reasons. Statistical significance between the *prhP* mutant (RQ5649) and the wild‐type strain (RK5050) was assessed using a post hoc Dunnett test following ANOVA. Significance level: ** indicates *P* < 0.01.

The T3SS is essential for pathogenicity of *R. solanacearum* (Genin and Denny, 2012; Valls *et al*., 2006), and the ability to degrade SA and FA can facilitate its infection process toward host plants (Lowe *et al.*, 2015; Lowe‐Power *et al.*, 2016). We ascertained whether PrhP is required for the infection process of *R. solanacearum* towards host plants. When tomato plants were challenged with the petiole‐inoculation, the *prhP* mutant killed approximately 75% of test plants at 10 dpi, which is significantly less virulent than the wild‐type strain (Fig. 4a). With the soil‐soaking inoculation, the *prhP* mutant eventually killed about half the test tomato plants by 25 dpi, which is significantly less virulent than the wild‐type strain (Fig. 4b). It is worthwhile noting that the *prhP* mutant exhibited much less virulence in soil‐soaking inoculated tomato plants compared with those with petiole inoculation (Fig. 4a,b). Moreover, the *prhP* mutant exhibited significantly impaired virulence in tobacco plants with both soil‐soaking and leaf‐infiltration inoculation methods (Fig. 4c,d). The complementary PrhP_OE1‐1_ fully restored the impaired virulence of the *prhP* mutant to that of the wild‐type strain in both tomato and tobacco plants (Fig. 4a,b,c,d), confirming that PrhP can greatly promote the infection process of *R. solanacearum* toward host plants.

The ability to degrade SA and FA facilitates the infection process of *R. solanacearum* toward host plants (Lowe *et al.*, 2015; Lowe‐Power *et al.*, 2016). We therefore generated a mutant with deletion of both *fcs* and *nag* operons (RQ6027) to ascertain whether the contribution of PrhP to pathogenicity depends on the degradation of SA and FA. The *fcs* and *nag* mutants (RQ6027) exhibited slightly less virulence than the wild‐type strain (RK5050), while the *prhP* mutant exhibited significantly less virulence than RQ6027 in tomato and tobacco plants regardless of inoculation method (Fig. 4a–d), indicating that PrhP greatly promotes the infection process of *R. solanacearum* as well as the degradation of SA and FA*.*

### PrhP regulates the T3SS mediated with HrpB but through some novel pathway

**Figure 5:**
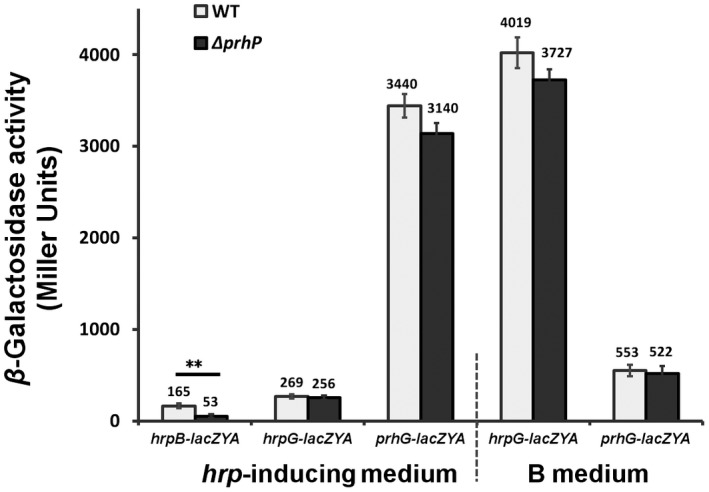
Expression of *hrpB‐lacZYA*, *hrpG‐lacZYA* and *prhG‐lacZYA* with *prhP* deletion. *Ralstonia solanacearum* cells were grown in nutrient‐rich or *hrp*‐inducing media to an OD_600_ of about 0.1 and subjected to enzyme assay. Mean values from all four biological replicates were averaged and presented with SD (error bars). Statistical significance between the *prhP* mutant (Δ*prhP*) and parent strains (WT) was assessed using a post hoc Dunnett test following ANOVA. Significance level: ** indicates *P* < 0.01.

In *R. solanacearum*, the T3SS and T3Es are directly controlled by the key regulator HrpB, and two close paralogues of HrpG and PrhG positively regulate *hrpB* expression in a parallel way (Mukaihara *et al.*, 2010; Plener *et al.*, 2010; Zhang *et al.*, 2013). We deleted *prhP* from reporter strains of RK5046 (*hrpB‐lacZYA*), RK5120 (*hrpG‐lacZAY*) and RK5212 (*prhG‐lacZAY*) to ascertain how PrhP regulates the T3SS expression. The *hrpB* expression was significantly impaired with *prhP* deletion (53 versus 165 Miller units of RK5046) in *hrp*‐inducing medium, which was activated in *hrp*‐inducing medium, while expression of the *hrpG* and *prhG* was not altered with *prhP* deletion in either nutrient‐rich or nutrient‐limited (*hrp*‐inducing) media (Fig. 5), indicating that regulation of PrhP on the T3SS is mediated with the key regulator HrpB, but through some novel pathway to HrpB.

### PrhP is important for expression of a subset of T3Es but not for hypersensitive response (HR) elicitation

Deletion of *prhP* significantly impaired *hrpB* expression, which directly controls expression of abundant T3Es in *R. solanacearum*. We therefore ascertained whether expression of T3Es was impaired with *prhP* deletion. The wild‐type strain (RK5050) and the *prhP* mutant (RQ5649) were cultivated in *hrp*‐inducing medium to an OD_600_ of 0.1 and total RNA was extracted for subsequent qRT‐PCR. In this study, a total of 11 T3Es of RipAA, RipAR, RipB, RipD, RipE1, RipO, RipP2, RipR, RipTAL, RipW and RipX (PopA, positive control) were selected for quantification of mRNA levels with qRT‐PCR. The qRT‐PCR results showed that expression levels of all these T3Es were significantly or distinctly impaired with *prhP* deletion (Fig. S1), confirming that PrhP is important for expression of the T3SS and a subset of T3Es in *R. solanacearum.*

GMI1000 elicits HR in tobacco leaves, and several T3Es are experimentally validated to be responsible for the HR elicitation of GMI1000 in tobacco leaves (Peeters *et al.*, 2013; Poueymiro *et al.*, 2009). Expression of a subset of T3Es was significantly impaired with *prhP* deletion and we further ascertained whether PrhP affects the HR elicitation of GMI1000 in tobacco leaves. Tobacco leaves (*Nicotiana tabacum* 'Bright Yellow') were infiltrated with a cell suspension at a concentration of 0.1 OD_600_ and development of necrotic lesions was investigated periodically. The *prhP* mutant (GF0018) exhibited similar development of the necrotic lesions as GF0001 (GMI1000, *popA‐lacZAY*) in tobacco leaves (Fig. S2), indicating that PrhP is not essential for the HR elicitation of GMI1000 in tobacco leaves.

### PrhP is important for the *in planta* growth of *R. solanacearum*

**Figure 6:**
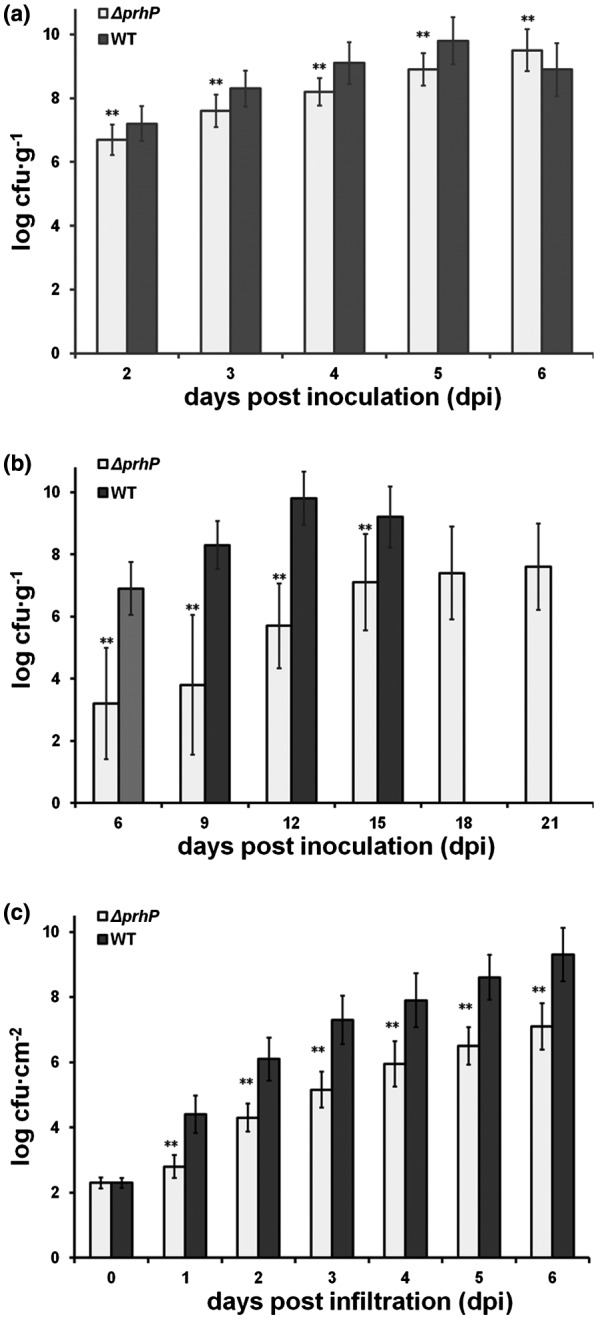
Bacterial growth in (a) tomato stems with petiole inoculation, (b) tomato stems with soil‐soaking inoculation and (c) tobacco leaves with leaf infiltration. With petiole inoculation, the wild‐type *Ralstonia solanacearum* strain (RK5050) normally causes tomato plants to wilt and die at about 3 and 6 days post‐inoculation (dpi), respectively. Bacterial cells were thus recovered from stems of petiole‐inoculated tomato plants at 2–6 dpi and subjected for quantification by dilution plating. With soil‐soaking inoculation, the wild‐type strain causes tomato plants to wilt and die at about 6 and 12 dpi, respectively. Cells were thus recovered from tomato stems at 6–12 dpi and subjected to quantification. The *prhP* mutant exhibited significantly less virulence on tomato plants, and bacterial cells were recovered from stems of the soil‐soaking inoculated tomato plants to 21 dpi. For leaf infiltration, about 50 µL of bacterial suspension at 10^4^ cfu/mL was infiltrated into tobacco leaves with a blunt‐end syringe. Cells were recovered daily from tobacco leaf disks to 6 dpi, when tobacco leaves became withered and dried. Mean values of at least four biological replicates were averaged and presented with SD (error bars). Statistical significance between the *prhP* mutant (RQ5649) and the wild‐type strain (RK5050) was assessed using a post hoc Dunnett test following ANOVA. Significance level: ** indicates *P* < 0.01.

Plants accumulate phenolic acids to form a defence against the invasion of pathogens, which inhibits the *in planta* growth of pathogens (Bellés *et al.*, 2006; Lowe‐Power *et al.*, 2018), while *R. solanacearum* can overcome this inhibition and proliferate extensively in the xylem vessels (Lowe *et al.*, 2015; Lowe‐Power *et al.*, 2016). The extensive proliferation *in planta* is one of the most important pathogenicity determinants in *R. solanacearum* (Genin and Denny, 2012), and we ascertained whether the *in planta* growth of *R. solanacearum* was altered with the *prhP* deletion. Petiole‐inoculated tomato plants normally start wilting at about 3 dpi and die at about 6 dpi. Bacterial cells were thus recovered from stems of the petiole‐inoculated tomato plants from 2 to 6 dpi and subjected for quantification with dilution plating. The *prhP* mutant (RQ5649) exhibited significantly impaired growth compared to the wild‐type strain (RK5050) in tomato stems by approximately one order of magnitude (Fig. 6a). The *prhP* mutant caused some of the soil‐soaking inoculated tomato plants to wilt and die at 10 and 25 dpi, respectively (Fig. 4b), and we ascertained the growth of the *prhP* mutant in stems of the soil‐soaking inoculated tomato plants to 21 dpi. Growth of the *prhP* mutant was significantly impaired in stems of the soil‐soaking inoculated tomato plants, which remained at levels of about 10^3^–10^4^ cfu/g to 9 dpi, and increased slowly to a maximum of about 10^9^ cfu/g at 21 dpi (Fig. 6b). It is worthwhile noting that growth of the *prhP* mutant in stems of the soil‐soaking inoculated tomato plants was significantly less than that in stems of the petiole‐inoculated tomato plants (Fig. 6a,b).

Tobacco plants exhibit different metabolic activities on SA from tomato plants (Bellés *et al.*, 2006, 1999), and we ascertained whether growth of *R. solanacearum* in tobacco plants was altered with *prhP* deletion. Tobacco leaves were infiltrated with cell suspension at a concentration of 10^4^ cfu/mL and the cell growth in tobacco leaves was quantified daily from 2 to 6 dpi, when tobacco leaves became withered and dried. Growth of the *prhP* mutant was also significantly impaired in tobacco leaves, but this was less than in the wild‐type strain by about one to two orders of magnitude (Fig. 6c). All these results confirm that PrhP is important for the *in planta* growth of *R. solanacearum* regardless of host plant species.

### PrhP regulates expression of a large set of virulence‐related genes

**Figure 7:**
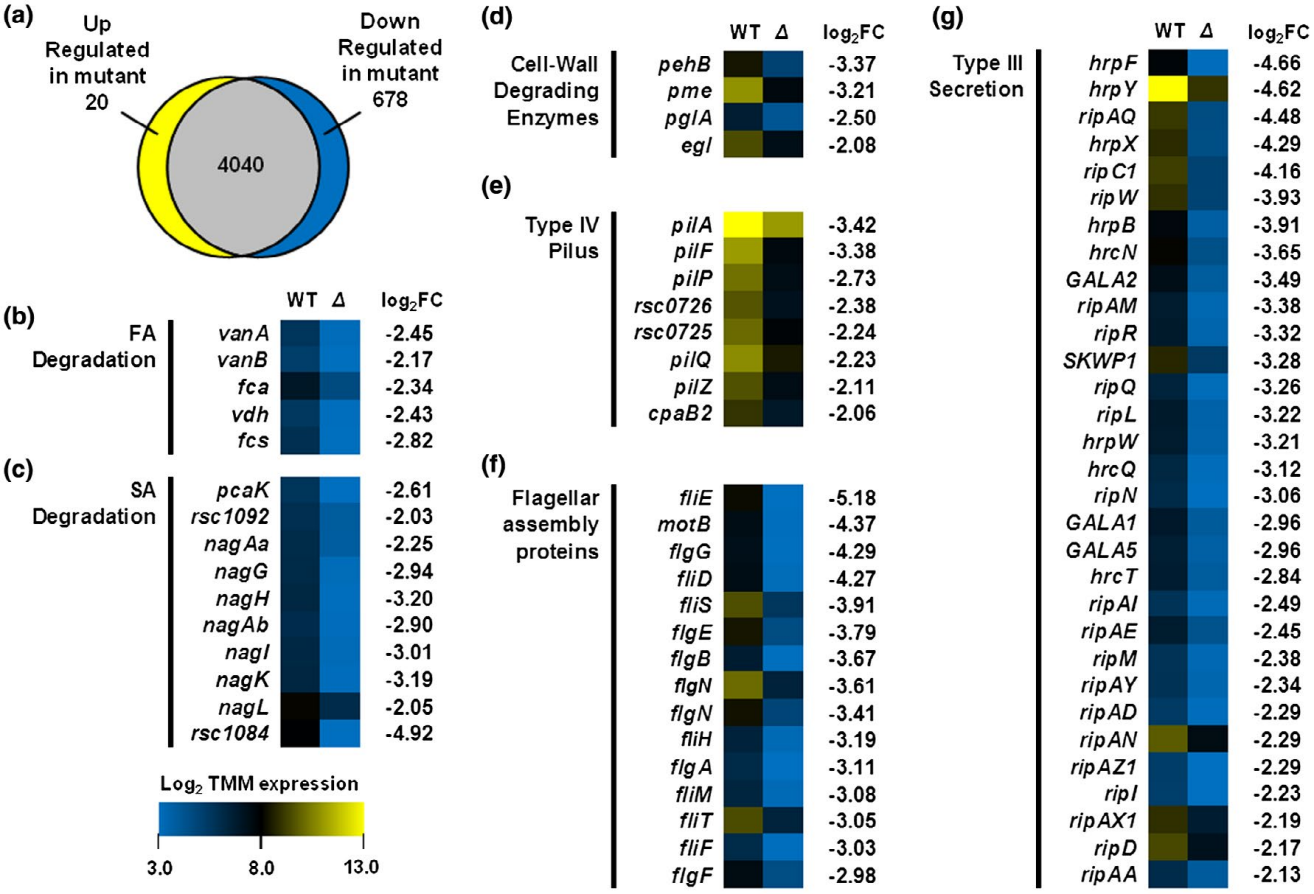
Gene expression in the *Ralstonia solancearum* *prhP* mutant with RNA‐seq. Briefly, total RNAs were isolated from three biological replicates according to the TRIzol reagent method (Life Technologies, Carlsbad, CA, USA) and subjected to RNA‐seq. Mean values of three biological replicates were averaged and subjected to statistical analysis. (a) A proportional Venn diagram of expression patterns created using BioVenn. Genes with relative expression levels greater than 2‐fold different between the *prhP* mutant and the wild‐type strain and adjusted *P* values of <0.05 were classified as differentially expressed genes (DEGs). (b)–(g) Heat maps show absolute expression of DEGs in different functional categories of (b) ferulic acid (FA) degradation, (c) salicylic acid (SA) degradation, (d) cell wall‐degrading enzymes, (e) type IV pilus, (f) flagellar assembly proteins and (g) T3SS and T3Es. Heat maps indicating low absolute expression (blue; 3.0) to high absolute expression (yellow; 13.0) are shown to the right of gene names. The fold change (*prhP* mutant versus RK5050) is shown to the right of each heat map.

*Ralstonia solanacearum* integrates numerous virulence factors to develop an infection process toward host plants, including the flagella, type IV pili, EPS and CWDEs (Genin and Denny, 2012; Hikichi *et al.*, 2007). PrhP greatly promotes the infection process of *R. solanacearum* besides the degradation of SA and FA, and we profiled a transcriptome analysis to address its multiple roles in the pathogenicity. The transcriptomic analysis revealed that a total of 698 genes were differentially expressed by more than 2‐fold between the wild‐type strain (RK5050) and the *prhP* mutant (RQ5649), of these 20 and 678 genes were up‐ and down‐regulated, respectively (Fig. 7a).

Consistent with the above results from the promoter activity assay, the expression level of *nagAa* was significantly down‐regulated by 4.76‐fold in the *prhP* mutant (Fig. 7c). The *nag* operon contains 10 genes, which are responsible for SA degradation (Lowe‐Power *et al.*, 2016). Expression of all these genes was significantly down‐regulated in the *prhP* mutant (Fig. 7c). Operons of *fca‐vdh‐fcs* and *vanAB* are responsible for FA degradation (Lowe *et al.*, 2015). Expression of all these genes was significantly down‐regulated in the *prhP* mutant (Fig. 7b).

The T3SS is directly controlled by the key regulator HrpB and globally regulated with a complex network, including dozens of regulators (Hikichi *et al.*, 2017; Vasse *et al.*, 1995). Expression of *hrpB* was indeed significantly down‐regulated in the *prhP* mutant by 15.03‐fold, but no alteration in expression of *prhA*, *prhI/R*, *hrpG*, *prhG*, *prhN*, *xpsR* and *phcA* with more than 4‐fold in the *prhP* mutant (Fig. 7g). It is consistent with our above results from the promoter activity assay in respective reporter strains that PrhP positively regulates the T3SS mediated with the key regulator HrpB but through some novel pathway. The *hrp* gene cluster contains approximately 20 genes, and expression levels of 12 of these genes were significantly down‐regulated in the *prhP* mutant (Fig. 7g). Moreover, the HrpB directly controls expression of a great number of T3Es, and expression of most of the T3Es was significantly down‐regulated in the *prhP* mutant (Fig. 7g), which is consistent with the above results from the qRT‐PCR.

In addition, expression of many genes, encoding most of the components for the flagellum, was significantly down‐regulated in the *prhP* mutant (Fig. 7f), indicating that the flagellum assembly is impaired with *prhP* deletion. The flagella are known to be essential for bacterial swimming and we evaluated the impact of PrhP on swimming motility. Consistent with transcriptomic results, swimming halos produced by the *prhP* mutant were significantly smaller than those of the wild‐type strain on semi‐solid motility agar plates (Fig. S3). Expression of many genes related to type IV pili, which are known to be important for attachment to roots and twitching motility, and contribute to pathogenicity, was significantly down‐regulated with *prhP* deletion (Fig. 7e). Expression of most of the genes related to the type IV pili was significantly down‐regulated in the *prhP* mutant except three genes of *cpaB*, *pilM* and *rsp1292*, expression of which was up‐regulated by 2.1‐fold, 3.6‐fold and 2.7‐fold, respectively (Fig. 7e). Moreover, expression of several genes related to the CWDEs was significantly down‐regulated with *prhP* deletion (Fig. 7d), while expression of genes related to the EPS was slightly up‐regulated with *prhP* deletion (data not shown). All these results confirm that PrhP positively regulates expression of a large set of virulence‐related genes and promotes the infection process of *R. solanacearum* toward host plants.

## Discussion

In the present study, we provided multiple lines of evidence to demonstrate that PrhP, a novel PadR regulator, plays a positive role in the detoxification of phenolic acids and greatly promotes the infection process of *R. solanacearum* toward host plants. Phenolic acids are broadly antimicrobial and many bacterial species express PADses to convert these chemicals into less toxic derivatives for detoxification (Park *et al.*, 2017; Tran *et al.*, 2008). The *prhP* mutant was much more sensitive to SA and FA (representative phenolic acids) than the wild‐type strain, confirming that the PrhP is important for *R. solanacearum* to promote the detoxification of phenolic acids. This is consistent with the fact that PadR regulators usually promote detoxification of phenolic acids in many bacterial species (Nguyen *et al.*, 2011; Park *et al.*, 2017). The PadR regulators usually repress the expression of PADse genes in the absence of phenolic acids by binding to their promoters but initiate the expression of PADse genes in the presence of phenolic acids by release from their promoters (Lowe‐Power *et al.*, 2018; Nguyen *et al.*, 2011). Different from the PADse‐based detoxification in many bacterial species, *R. solanacearum* detoxifies SA and FA by metabolizing them as carbon sources (Lowe *et al.*, 2015; Lowe‐Power *et al.*, 2016). Degradation of SA and FA in *R. solanacearum* is experimentally validated to be dependent on *nag*‐encoding dioxygenases and *fcs*‐encoding feruloyl‐CoA, respectively (Lowe *et al.*, 2015; Lowe‐Power *et al.*, 2016). It is not contradictory that PrhP was demonstrated to be a novel positive regulator of *nag* expression even in the absence of SA based our results from the RNA‐seq and promoter assay since the *nag*‐encoding dioxygenases and *fcs*‐encoding feruloyl‐CoA are not PADse. Note that no PADse is annotated in numerous genomes of *R. solanacearum* strains, indicating that degradation of SA and FA is mainly dependent on operons of the *fcs* and *nag* in *R. solanacearum*. PrhP displays about 37% of amino acid identity with several well‐characterized PadR regulators, such as the AphA in *Vibrio cholerae* and LadR in *Lactobacillus plantarum* (De Silva *et al.*, 2005; Huillet *et al.*, 2006). Moreover, no conserved palindromic sequences can be found for the PadR binding in promoter regions of *fcs* and *nag* operons. The regulation mechanism of PrhP on the expression of *fcs* and *nag* remains to be further elucidated.

The *nag* expression was greatly enhanced with supplementary SA (Fig. 2), which was consistent with the fact that most of the PADses are substrate inducible (Huillet *et al.*, 2006, Lowe‐Power *et al.*, 2018; Van Duy *et al*., 2007). Although the *nagAa* expression was significantly impaired with *prhP* deletion in the presence of SA, it remained at a level of about 80% of the wild‐type strain, indicating that the *prhP* mutant should be able to degrade SA to some extent. This could explain our result that the *nag* mutant was much more sensitive than the *prhP* mutant to supplementary SA (Fig. 1a). We supposed that some novel regulators should regulate the expression of *fcs* and *nag* operons besides the PrhP. This speculation is also demonstrated in some published papers that *R. solanacearum* might harbour some *fcs*‐independent pathway for specific degradation of HCAs (Lowe *et al.*, 2015). We are currently screening dozens of candidates showing an impact on expression of *nagAa‐lacZYA* by transposon mutagenesis, and their transcriptional regulation on *nag* genes is under elucidation.

Phenolic acids play dual roles in the interaction between plants and plant pathogens to inhibit bacterial growth as antibiotics and also defend against pathogenic invasion by triggering an immune response, such as the SA‐triggered immune‐signalling pathways (Campos *et al.*, 2014; Tsuda and Katagiri, 2010). The ability to degrade phenolic acids could thus protect *R. solanacearum* from the toxicity of these chemicals, and also impair the triggered plant immune response (Lowe‐Power *et al.*, 2016, 2018). Supplementary SA and FA severely inhibited the growth of the *prhP* mutant in medium but at quite high concentrations, such as 500 μM of SA and 1500 μM of FA. Although it is complicated to measure concentrations of SA and FA in plants, several lines of evidence suggest that free SA can be accumulated to quite high concentrations in plant tissues that is capable to inhibit growth of *R. solanacearum* (Cameron and Zaton 2004; Huang *et al.*, 2006; Smith‐Becker *et al.*, 1998). Concentrations of some phenolic acids are also reported to be locally high in xylems of roots and stems where they are released by sentinel phenolic‐storing cells (Beckman, 2000; Wallis and Chen, 2012). In addition, bacterial growth is much more severely inhibited with the mixture of HCAs than those with limited compounds of HCAs (Harris *et al.*, 2010). PrhP should be involved in the detoxification of phenolic acids more than SA and FA, which could explain the result that the *fcs* and *nag* mutants exhibit faintly impaired growth in host plants (Lowe *et al.*, 2015; Lowe‐Power *et al.*, 2016), while the *prhP* mutant exhibits significantly impaired growth in host plants. Extensive proliferation in xylem vessels is one of the most important pathogenicity determinants in *R. solanacearum* (Cunnac *et al.*, 2004; Denny, 1995). The *fcs* and *nag* mutants exhibit faintly impaired virulence compared to the wild‐type strain (Lowe *et al.*, 2015; Lowe‐Power *et al.*, 2016), while the *prhP* mutant exhibits significantly less virulence compared to the wild‐type strain in host plants. The mutant lacking both *fcs* and *nag* exhibits slightly less virulence compared with the *fcs* and *nag* mutants, which is consistent with previous demonstration that the mixture of HCAs exhibits more a severe inhibitory effect on bacterial growth than the limited compounds of HCAs (Harris *et al.*, 2010). The *prhP* mutant is significantly less virulent than the *fcs* and *nag* mutants in tomato and tobacco plants, indicating that PrhP might be involved in the detoxification of more phenolic acids than SA and FA, or is important for some other pathogenicity determinants.

The T3SS is another essential pathogenicity determinant in *R. solanacearum* (Genin, 2010; Hikichi *et al.*, 2007) and is significantly impaired with *prhP* deletion both *in vitro* and *in planta*. To our knowledge, this is the first report on PadR regulators that positively regulates T3SS expression in pathogenic bacteria. The *hrpB* expression was also significantly impaired with *prhP* deletion, which is consistent with the fact that HrpB directly controls the entire T3SS and T3Es (Hikichi *et al.*, 2007; Valls *et al.*, 2006). PrhP positively regulates *hrpB* expression and in turn regulates the expression of T3SS and T3Es in *R. solanacearum*. *hrpB* expression is positively regulated by two close paralogues of HrpG and PrhG in a parallel way (Plener *et al.*, 2010; Zhang *et al.*, 2013), while no alteration was found in their expression with the *prhP* deletion, indicating that PrhP positively regulates *hrpB* expression through some novel pathway. As the OmpR/PhoB family of two‐component response regulators, HrpG and PrhG should respond to host signals by phosphorylation at some residues and then phosphorylated HrpG and PrhG begin to activate *hrpB* expression (Yoshimochi *et al*., 2009). It remains to be further elucidated whether PrhP is involved in host signal response with HrpG and PrhG. It is worthwhile noting that the HR elicitation of GMI1000 is not altered with *prhP* deletion in tobacco leaves, indicating that weakly expressed T3Es might be enough to trigger plant immunity since they are not completely diminished with *prhP* deletion. Moreover, our results from the RNA‐seq reveal that PrhP is involved in expression of a large set of virulence‐related genes, such as the flagella, type four pili and CWDEs. Expression of most of the genes for the flagellum assembly was significantly down‐regulated with *prhP* deletion, and the flagella‐mediated swimming motility was indeed impaired on semi‐solid motility agar plates with *prhP* deletion. The flagella, type 4 pili and CWDEs play important roles in adhesion to roots and cell wall destruction, which are especially important at the early stage of the infection process (Denny, 1995; Liu *et al.*, 2005; Tans‐Kersten *et al.*, 1998, 2004). Expression of many of these genes was significantly down‐regulated with *prhP* deletion, which could explain our results that the *prhP* mutants were much less virulent in soil‐soaking inoculated tomato plants than those with petiole inoculation. Tobacco plants exhibit different metabolic activities on phenolic acids, and different plants display different symptoms depending upon the infecting strains (Bellés *et al.*, 1999, 2006). For instance, the secondary metabolites in xylem vessels are revealed to be quite different between these two plants, including phenolic acids, saccharides and amino acids (Lowe‐Power *et al.*, 2018; Zuluaga *et al*., 2013). This might be a reason why the *prhP* mutants exhibit almost equally less virulence on tobacco plants regardless of inoculation method.

Taken together, our results from the growth assay, promoter assay, RNA‐seq and qRT‐PCR demonstrate that PrhP, a novel PadR regulator, plays an important regulatory role in multiple processes involved in the infection of *R. solanacearum*, such as the detoxification of phenolic acids, extensive proliferation in host plants and expression of a great number of virulence‐related genes. This is the first report on the PadR regulators that regulate the T3SS, which could improve our understanding of the various biological functions of PadR regulators and complex regulatory pathway on the T3SS in *R. solanacearum*.

## Experimental Procedures

### Bacterial strains and growth conditions

*Escherichia coli* strains of DH12S and S17‐1 were grown at 37 °C in LB medium for plasmid construction and conjugational transfer, respectively. *Ralstonia solanacearum* strains, listed in Table 1, were grown at 28 °C in nutrient‐rich medium (B medium) or nutrient‐limited medium (sucrose medium, *hrp‐*inducing medium) (Yoshimochi *et al.*, 2009).

**Table 1:** Bacterial strains used in this study

Strain	Relative characteristics	References
OE1‐1	Wild‐type, race 1, biovar 3	Kanda *et al*. (2003)
RK5046	OE1‐1, *hrpB‐lacZYA*	Yoshimochi *et al*. (2009)
RK5050	OE1‐1, *popA‐lacZYA*	Yoshimochi *et al*. (2009)
RK5120	OE1‐1, *hrpG‐lacZYA*	Yoshimochi *et al*. (2009)
RK5212	OE1‐1, *prhG‐lacZYA*	Zhang *et al.* (2013)
RQ5649	*popA‐lacZYA*, Δ*prhP*	This study
RQ5625	RK5050, Δ*fcs*	This study
RQ5651	RK5046, *ΔprhP*	This study
RQ5657	RK5212, Δ*prhP*	This study
RQ5660	RK5120, Δ*prhP*	This study
RQ6207	RQ5625, Δ*nagAL*	This study
RQ6058	RK5050, Δ*nagAL*	This study
RQ6061	OE1‐1, Δ*prhP*	This study
RQC380	RQ5649, +*prhP_OE1‐1_*	This study
RQC607	OE1‐1, *nagAa‐lacZYA*	This study
RQC609	RQ6061, *nagAa‐lacZYA*	This study
GMI1000	Wild‐type, race 1, biovar 4	Salanoubat *et al*. (2002)
GF0001	GMI1000, *popA‐lacZYA*	Zhang *et al.* (2018)
GF0018	GF0001, Δ*prhP*	This study
RQC089	GF0018, +*prhP_OE1‐1_*	This study

### Construction of *prhp* in‐frame deleted mutants

Mutants with in‐frame deletion of target genes were generated with pK18mobsacB‐based homologue recombination (Zhang *et al.*, 2015). In general, two DNA fragments flanking the *prhP* were conjugated with joint PCR and subcloned into pK18mobsacB to generate pK18d0309. After validating the sequence, pK18d0309 was transferred into *R. solanacearum* by conjugation with S17‐1. The *prhP* mutants were generated (listed in Table 1) and confirmed by colony PCR with primer pairs of 0309A1B and 0309B2H. The primers used in this study are listed in Table S1.

### Complementation analyses

Complementation analyses were performed in this study with the pUC18‐mini‐Tn*7*T‐Gm‐based site‐specific chromosome integration system (Choi *et al.*, 2005; Zhang *et al.*, 2015). In general, the *prhP* gene and its upstream region of about 600 bp, empirically harbouring the native promoter, was PCR amplified and finally cloned into pUC18‐mini‐Tn7T‐Gm to get pUCprhP. After validating the sequence, complementary *prhP* was integrated into *R. solanacearum* chromosome at a single *att*Tn*7* site (25 bp downstream of *glmS*) and confirmed by colony PCR with primers of glmsdown‐Tn7R (Zhang *et al.*, 2011).

### Construction of reporter strains with *nagAa‐laZYA* for promoter activity assay

The *nagAa‐lacZYA* reporter strains were generated with the pUC18‐mini‐Tn*7*T‐Gm‐based site chromosome integration system. In general, promoterless *lacZYA* was fused to *nagAa* at 54 bp after the start codon, in which 6 bp of nucleotide acids were replaced into *Kpn*I by PCR for *lacZYA* insertion. A DNA fragment containing the promoter region and the *Kpn*I site was first cloned into pUC18‐mini‐Tn*7*T‐Gm and then *lacZYA* was inserted to generate pUCnagAa‐lacZYA. After validating the sequence, this reporter fusion was integrated into the chromosome of the wild‐type strain and the *prhP* mutant to get the desired mutants (listed in Table 1).

### β‐Galactosidase assay

The β‐galactosidase assay was performed to evaluate expression levels of *lacZYA*‐fused genes. The enzyme assay *in vitro* was expressed in Miller units (Miller, 1992), and that *in planta* was normalized with luminescence divided by cells number (Zhang *et al.*, 2013). Each assay was repeated for four independent experiments with four replications per trial. The mean values of all the experiments were averaged with SD and the statistical significance was assessed using a post hoc Dunnett test following ANOVA.

### Virulence assay and HR test

The virulence assay and HR test were performed as previously described (Yao and Allen, 2007). Wilt‐susceptible tomato plants (*Solanum lycopersicum* 'Moneymaker') and tobacco plants (*N. tabacum* 'Bright Yellow') were subjected to virulence assay with soil‐soaking inoculation method, which mimics natural invasion through roots, and petiole inoculation, which enables bacteria to directly invade xylems vessels. Each assay was repeated for four independent experiments with 12 plants per trial. Wilt symptoms of plants were inspected as 1–4 disease index and mean values of all experiments were averaged with SD. Whereas the SD was not presented in figures for virulence assay due to the consideration of aesthetic appearance. The statistical significance was assessed using a post hoc Dunnett test following ANOVA. The HR test was carried out on tobacco leaves of *N. tabacum* with leaf infiltration and the symptom development of HR was recorded periodically. Each test was repeated independently at least four times with six plants per trial and a representative result is presented.

### Bacterial growth assay

Bacterial growth *in planta* was quantified by dilution plating (Zhang *et al.*, 2013) and that in medium was assessed with OD_600_. Each assay was repeated for at least four independent experiments with three replications per trial*.* Mean values of all experiments were averaged with SD and statistical significance was assessed using a post hoc Dunnett test following ANOVA.

### RNA extraction and deep sequencing

*Ralstonia solanacearum* strains were grown in *hrp‐*inducing medium to OD_600_ of about 0.1 and total RNA was isolated with TRIzol reagent method according to the manufacturer’s instructions (Life Technologies, Carlsbad, CA, USA). Total RNAs were isolated with three biological replicates and subjected to RNA‐seq. After validating the quality, RNA samples were entrusted to Shanghai Biozeron Biotechnology Co., Ltd (Shanghai, China) for deep sequencing. In general, contaminative genomic DNA was removed using RQ DNase I (Promega) and ribosomal RNA was removed using a Ribo‐Zero rRNA Removal Kit (Gram‐negative bacteria, Illumina, Madison, WI, USA). The cDNA synthesis, end repair and ligation of the Illumina adaptors were performed according to Illumina’s protocol, and subjected to RNA‐seq using Illumina HiSeq PE 2 × 151‐bp read length, resulting in *c*.10 million reads per sample.

Raw reads were trimmed and quality controlled by Trimmomatic software with default parameters (http://www.usadellab.org/cms/uploads/supplementary/Trimmomatic), and clean reads were separately aligned to the reference genome with orientation mode using hisat2 (https://ccb.jhu.edu/software/hisat2/index.shtml) software. The expression levels of each transcript were calculated using the fragments per kilobase of exon per million mapped reads to identify differential expression of genes between two different samples and cuffdiff (http://cufflinks.cbcb.umd.edu/) was used for differential expression analysis. The mean values of three biological replicates were averaged and subjected for statistical analysis.

### qRT‐PCR analysis

cDNA was synthesized using the PrimeScript RT Reagent Kit with gDNA Eraser (Perfect for Real Time, Takara, Japan) according to the manufacturer’s instructions (contaminated genome DNA could be removed by the gDNA Eraser in this kit). The One Step SYBR PrimeScript PLUS RT‐PCR Kit (Takara, Dalian, China) was used for qRT‐PCRs with the Applied Biosystems 7500 Real‐Time PCR System. Primers used in this study were selected as previously described and *serC* gene was used as the reference gene for normalization of gene expression (Monteiro *et al.*, 2012; Wu *et al.*, 2015). Each assay was repeated from RNA isolation for at least three independent experiments with four replications per trial. The mean values of all experiments were averaged with SD, and the statistical significance between the wild‐type strain and the *prhP* mutant was assessed using a post hoc Dunnett test following ANOVA.

## Supporting information


**Fig. S1** Relative expression of T3Es genes in the *prhP* mutant. Strains were grown in *hrp‐*inducing medium to an OD_600_ of about 0.1 and total RNA was isolated. The cDNA was synthesized using the PrimeScript RT Reagent Kit with gDNA Eraser and mRNA levels of representative T3Es genes were determined by qRT‐PCR with reference gene as* serC* for normalization. Normalized values of the *prhP* mutant were divided with those of wild‐type (WT) strain and relative values (relative expression) were presented. Mean values of at least three biological replicates were averaged and presented with SD (error bars). Statistical significance was assessed between *prhP* mutants and WT strain. Significance level: * indicates *P* < 0.05 and ** indicates *P* < 0.01.

**Fig. S2** HR test. Approximate 50 μL of bacterial suspension at 10^8^ cfu/mL was infiltrated into tobacco leaves with a blunt‐end syringe: (A) GF001 (GMI1000, *popA‐lacZYA*), (B) GF0018 (GF0001, Δ*prhP*) and (C) distilled water. Development of necrotic lesions was observed periodically and pictures were taken. Each experiment was repeated at least four times and each treatment contained four plants. The results presented are from a representative experiment, and similar results were obtained in all experiments.

**Fig. S3** Swimming motility of* prhP* mutants. Bacterial suspension (3 μL) at OD_600_ of 1.0 was dropped onto 0.3% agar plates and kept at 28 °C for 48 h. Swimming motility was quantified as halo diameters in millimetres. Mean values of three biological replicates with four replicates per trial were averaged and presented with SD (error bars). Statistical significance was assessed between RQ5649 and RK5050. Significance level: ** indicates *P* < 0.01.

**Table S1** Primers used in this study.
