# Supplementary material for: Involvement of a PadR regulator PrhP on virulence of Ralstonia solanacearum by controlling detoxification of phenolic acids and type III secretion system
Source: Mol Plant Pathol. 2019 Aug 8;20(11):1477–90. doi: 10.1111/mpp.12854 (PMC6804342; doi:10.1111/mpp.12854)
Supplement: Supplementary file 1 — Fig. S1 Relative expression of T3Es genes in the prhP mutant. Strains were grown in hrp‐inducing medium to an OD600 of about 0.1 and total RNA was isolated. The cDNA was synthesized using the PrimeScript RT Reagent Kit with gDNA Eraser and mRNA levels of representative T3Es genes were determined by qRT‐PCR with reference gene as serC for normalization. Normalized values of the prhP mutant were divided with those of wild‐type (WT) strain and relative values (relative expression) were presented. Mean values of at least three biological replicates were averaged and presented with SD (error bars). Statistical significance was assessed between prhP mutants and WT strain. Significance level: * indicates P < 0.05 and ** indicates P < 0.01. [file MPP-20-1477-s001.docx]

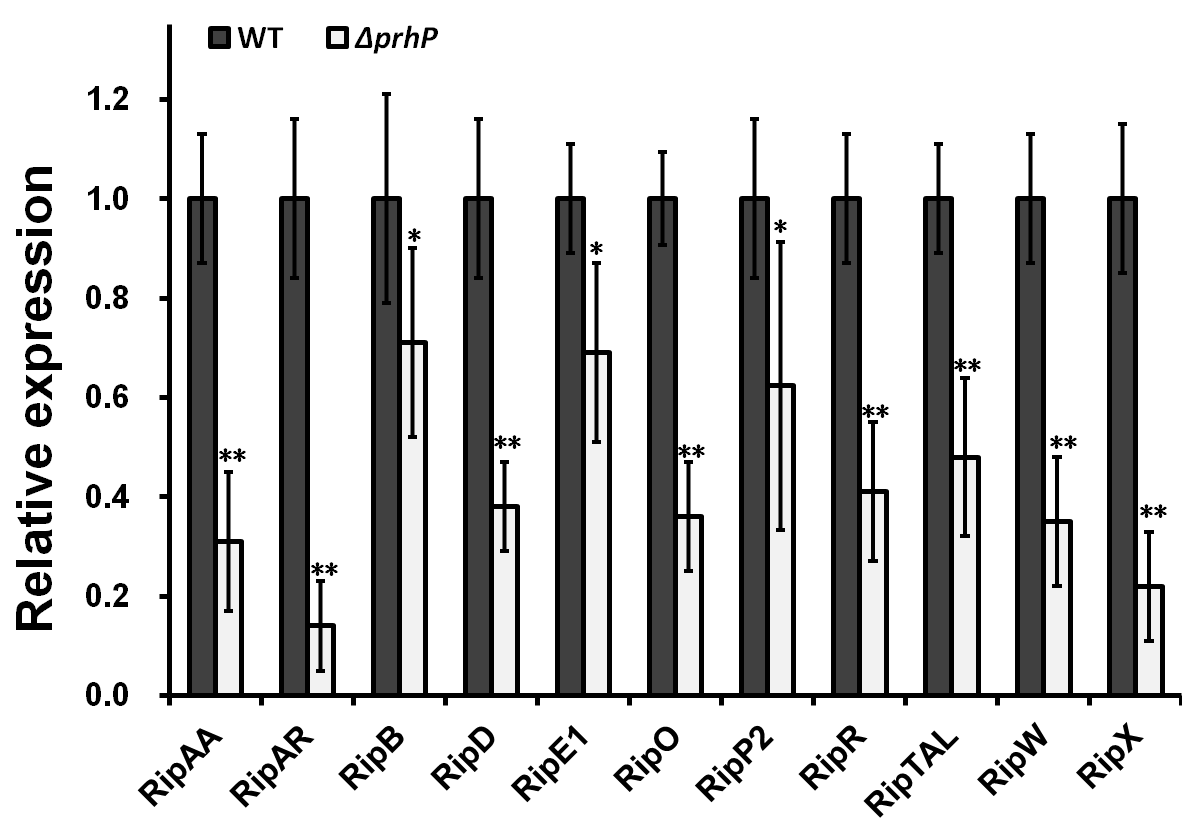


Fig. S1. Relative expression of T3Es genes in *prhP* mutant. Strains were grown in *hrp-*inducing medium to an OD600 as about 0.1 and total RNA was isolated. The cDNA was synthesized using the PrimeScript™ RT Reagent Kit with gDNA Eraser and mRNA levels of representative T3Es genes were determined by qRT-PCR with reference gene as *serC* for normalization. Normalized values of *prhP* mutant were divided with those of wild type strain (WT) and relative values (relative expression) were presented. Mean values of at least three biological replicates were averaged and presented with SD (error bars). Statistical significance was assessed between *prhP* mutants and wild type strain. Significance level, * indicates *P*＜0.05 and ** indicates *P*＜0.01.
