# Supplementary material for: Involvement of a PadR regulator PrhP on virulence of Ralstonia solanacearum by controlling detoxification of phenolic acids and type III secretion system
Source: Mol Plant Pathol. 2019 Aug 8;20(11):1477–90. doi: 10.1111/mpp.12854 (PMC6804342; doi:10.1111/mpp.12854)
Supplement: Supplementary file 2 — Fig. S2 HR test. Approximate 50 μL of bacterial suspension at 108 cfu/mL was infiltrated into tobacco leaves with a blunt‐end syringe: (A) GF001 (GMI1000, popA‐lacZYA), (B) GF0018 (GF0001, ΔprhP) and (C) distilled water. Development of necrotic lesions was observed periodically and pictures were taken. Each experiment was repeated at least four times and each treatment contained four plants. The results presented are from a representative experiment, and similar results were obtained in all experiments. [file MPP-20-1477-s002.docx]

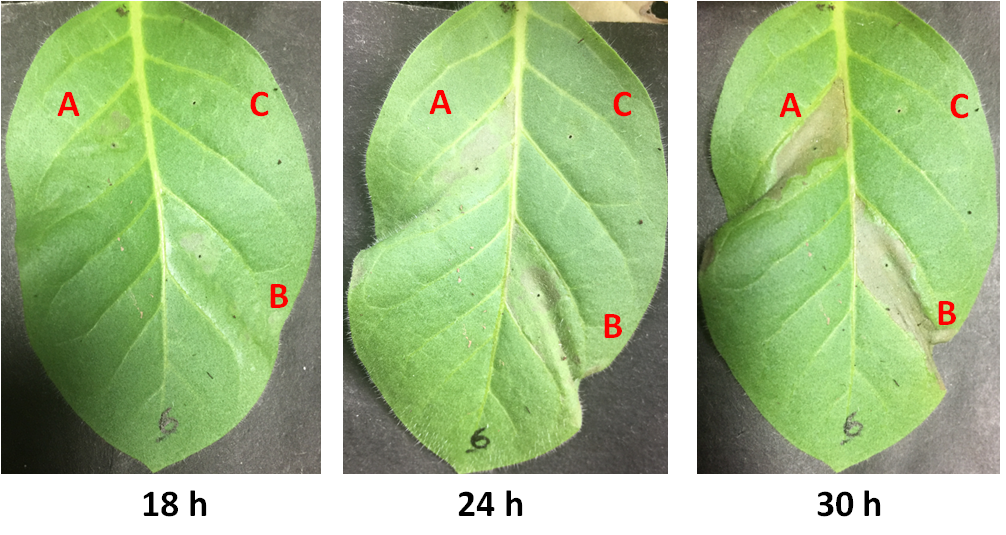


Fig. S2. HR test. Approximate 50 μl of bacterial suspension at 10^8^ cfu ml^-1^ was infiltrated into tobacco leaves with a blunt-end syringe. (A) GF001 (GMI1000, *popA-lacZYA*); (B) GF0018 (GF0001, *ΔprhP*); (C) distilled water. Development of necrotic lesions was observed periodically and pictures were taken. Each experiment were repeated at lest for four times and each treatment contains four plants. The results presented are from a representative experiment, and similar results were obtained in all experiments.
