# Supplementary material for: Involvement of a PadR regulator PrhP on virulence of Ralstonia solanacearum by controlling detoxification of phenolic acids and type III secretion system
Source: Mol Plant Pathol. 2019 Aug 8;20(11):1477–90. doi: 10.1111/mpp.12854 (PMC6804342; doi:10.1111/mpp.12854)
Supplement: Supplementary file 3 — Fig. S3 Swimming motility of prhP mutants. Bacterial suspension (3 μL) at OD600 of 1.0 was dropped onto 0.3% agar plates and kept at 28 °C for 48 h. Swimming motility was quantified as halo diameters in millimetres. Mean values of three biological replicates with four replicates per trial were averaged and presented with SD (error bars). Statistical significance was assessed between RQ5649 and RK5050. Significance level: ** indicates P < 0.01. [file MPP-20-1477-s003.docx]

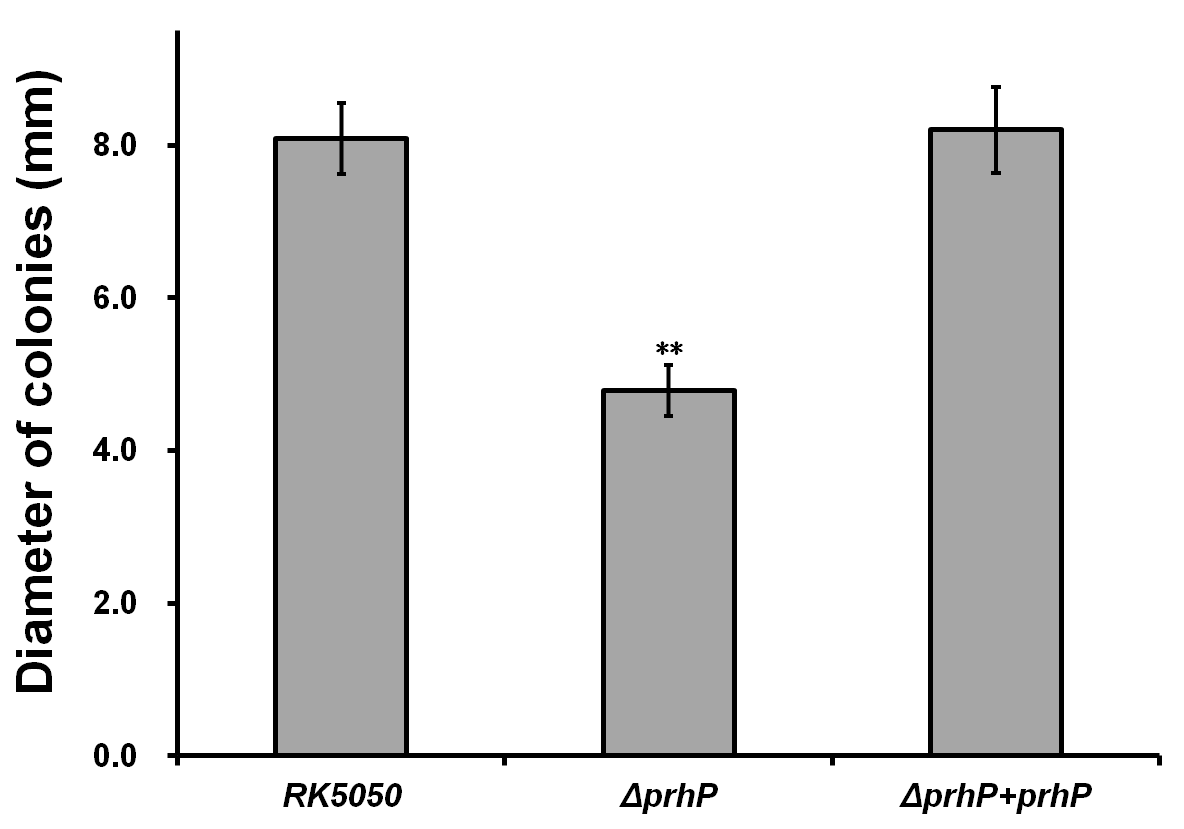


Fig. S3. Swimming motility of *prhP* mutants. Bacterial suspension (3 μl) at an OD600 of 1.0 was dropped onto 0.3 % agar plates and kept at 28°C for 48h. Swimming motility was quantified as halo diameters in mm. Mean values of three biological replicates with four replicates per trial were averaged and presented with SD (error bars). Statistical significance was assessed between RQ5649 and RK5050. Significance level, ** indicates *P*＜0.01.
