# Supplementary material for: Involvement of a PadR regulator PrhP on virulence of Ralstonia solanacearum by controlling detoxification of phenolic acids and type III secretion system
Source: Mol Plant Pathol. 2019 Aug 8;20(11):1477–90. doi: 10.1111/mpp.12854 (PMC6804342; doi:10.1111/mpp.12854)
Supplement: Supplementary file 4 — Table S1 Primers used in this study. [file MPP-20-1477-s004.docx]

**Table S1. Primers used in this study**

| primer | sequence |
| --- | --- |
| rsp0309A1B | CATGGATCCACCACATCGCGCGAGGGCAAC |
| rsp0309B1C | AATGCCCAGACGGGCTCCGGCCGCAAGTAATAGCAAGCAACAATCG |
| rsp0309A2C | CGATTGTTGCTTGCTATTACTTGCGGCCGGAGCCCGTCTGGGCATT |
| rsp0309B2H | CATAAGCTTCACCTTCGTGTTCGGCAACGC |
| rsp0309B3H | CATAAGCTTCAGGCCGGAACGGAGGCCGGCT |
| fcsA1B | AGCGGATCCGGCATTTGGTGGACG |
| fcsB1C | CGCCAGCATCACCTTGCCGTGACAGCGACCTCGCATCAGA |
| fcsA2C | TCTGATGCGAGGTCGCTGTCACGGCAAGGTGATGCTGGCG |
| fcsB2H | ctgAAGCTTcctcaagagctacgcgca |
| nagALA1E | GCGGAATTCGCAACGTATCGGCGTGGTT |
| nagALB1C | GCTGGGTTGTGGGCTCGCCGGGCCAGTGTCTCCGGGCTGG |
| nagALA2C | CCAGCCCGGAGACACTGGCCCGGCGAGCCCACAACCCAGC |
| nagALB2X | CGTCTAGAGTGGCCCAGAACAGCATGG |
| nagAaA1H | GCAAGCTTCCGCGACGATCGACACA |
| nagAaB2K | GCGGTACCGCTGACGATGCGCTGCAGC |
| glmsdown | GCGCTCAAGCTCAAGGAGATC |
| Tn7R | CACAGCATAACTGGACTGATTTC |
| lacZR1 | GCGCCATTCGCCATTCAGGCT |
